# Variegated Transcription of the WC1 Hybrid PRR/Co-Receptor Genes by Individual γδ T Cells and Correlation With Pathogen Responsiveness

**DOI:** 10.3389/fimmu.2018.00717

**Published:** 2018-05-07

**Authors:** Payal Damani-Yokota, Janice C. Telfer, Cynthia L. Baldwin

**Affiliations:** ^1^Program in Molecular and Cellular Biology, University of Massachusetts, Amherst, MA, United States; ^2^Department of Veterinary and Animal Sciences, University of Massachusetts, Amherst, MA, United States

**Keywords:** γδ T cells, co-receptors, WC1, T cell receptor, pattern recognition receptors

## Abstract

γδ T cells have broad reactivity and actively participate in protective immunity against tumors and infectious disease-causing organisms. In γδ-high species such as ruminants and other artiodactyls many γδ T cells bear the lineage-specific markers known as WC1. WC1 molecules are scavenger receptors coded for by a multigenic array and are closely related to SCART found on murine γδ T cells and CD163 found on a variety of cells. We have previously shown that WC1 molecules are hybrid pattern recognition receptors thereby binding pathogens as well as signaling co-receptors for the γδ T cell receptor. WC1^+^ γδ T cells can be divided into two major subpopulations differentiated by the WC1 genes they express and the pathogens to which they respond. Therefore, we hypothesize that optimal γδ T cell responses are contingent on pathogen binding to WC1 molecules, especially since we have shown that silencing WC1 results in an inability of γδ T cells from primed animals to respond to the pathogen *Leptospira*, a model system we have employed extensively. Despite this knowledge about the crucial role WC1 plays in γδ T cell biology, the pattern of WC1 gene expression by individual γδ T cells was not known but is critical to devise methods to engage γδ T cells for responses to specific pathogens. To address this gap, we generated 78 γδ T cell clones. qRT-PCR evaluation showed that approximately 75% of the clones had one to three WC1 genes transcribed but up to six per cell occurred. The co-transcription of WC1 genes by clones showed many combinations and some WC1 genes were transcribed by both subpopulations although there were differences in the overall pattern of WC1 genes transcription. Despite this overlap, *Leptospira*-responsive WC1^+^ memory γδ T cell clones were shown to have a significantly higher propensity to express WC1 molecules that are known to bind to the pathogen.

## Introduction

WC1 family members are T cell co-receptors and pathogen recognition receptors uniquely expressed by the majority of γδ T cells in the blood of ruminants (1). Structurally they are Group B scavenger receptor cysteine rich (SRCR) molecules (2) and belong to the CD163 family (2, 3) which also includes the murine γδ T cell marker SCART (3, 4). WC1-expressing γδ T cells in cattle, a γδ T cell high species and our experimental model, are classified into two main subpopulations referred to as WC1.1^+^ or WC1.2^+^ based on expression of different WC1 molecules that react with monoclonal antibodies (mAb) that recognize epitopes in their N-terminal SRCR domains (5). We and others have previously shown that these subpopulations also differ in their responses to pathogens. For example, following *in vivo* priming of cattle cells in the WC1.1^+^ subpopulation respond by proliferation and interferon-γ production to *Leptospira* spp. in *in vitro* recall responses (6, 7) whereas cells in the WC1.2^+^ subpopulation respond *in vitro* to other pathogens such as *Anaplasma marginale* following infection (8). When cattle are infected with virulent strains of *Mycobacterium bovis* both WC1^+^ lineages are recruited to the granulomas in infected cattle (9) but only the WC1.1^+^ cells respond to the vaccine strain BCG (10). Following *in vivo*-sensitization, these WC1^+^ mycobacterial-responsive bovine γδ T cells also have been shown to respond in recall responses *in vitro* to both protein and non-protein antigens while WC1^+^ and CD8^+^ γδ T cells respond to BCG-infected macrophages (9, 11). Adaptive-like memory γδ T cells are not confined to the bovine model having been described for specific subpopulations of murine γδ T cells (12, 13) and to be sensitized by *Listeria monocytogenes* (14) and *Staphyloccus aureus* (15) *in vivo* while in humans and non-human primates memory γδ T cells responses to mycobacteria (16–18), influenza (19), and malaria (20) have been reported.

The 13 WC1 molecules can be divided into 10 WC1.1-types and 3 WC1.2-types based on signature insertions or deletions of amino acids in their most membrane-distal SRCR domain known as the a1 domain (Figure S1 in Supplementary Material). The first sequenced WC1 genes (21) and therefore considered to be the archetypal WC1.1 [coded for by *WC1-3* (22)] and WC1.2 molecules [coded for by *WC1-4* (22)] differ in their binding to *Leptospira*. As many as five of WC1-3’s 11 SRCR domains are engaged, including the a1 domain, while none of the SRCR domains of its WC1.2 counterpart, WC1-4, bind *Leptospira* despite considerable sequence similarity (23). Binding can be disrupted by a single amino acid mutation (23). Four other WC1.1 type molecules also have SRCR domains that bind *Leptospira* but none of the WC1.2 molecules have such domains. This knowledge contributed to our understanding of the dichotomy in the ability of cells in the subpopulations to respond to particular pathogens. We hypothesize that the WC1 molecules expressed by a γδ T cell contribute to its pathogen responsiveness and that co-expression of multiple WC1 gene products that bind the same pathogen could result in increased avidity for the pathogen and amplify the signal in a dose-dependent manner (i.e., the more WC1’s of the same or multiple types that bind the pathogen, the stronger the cellular activation signal). The latter is based on our findings that when all (vs. a proportion of) the WC1 receptors are co-crosslinked in conjunction with the γδ T cell receptor (TCR) augmentation of the cellular activation is enhanced (24). Conversely, when WC1 expression is downregulated by RNA silencing there is an abrogation of γδ T cell response (25).

The previous analyses of WC1 transcripts in the subpopulations suggest co-expression of WC1 genes in a variegated pattern. While the WC1.2^+^ population has transcripts for all three WC1.2-type genes, only two of the three WC1.2-type gene products react with the population-defining mAb (5, 26). This suggests the non-mAb-reactive gene product is co-expressed in cells expressing the mAb-reactive gene product. WC1.3^+^ cells, a subpopulation of the WC1.1^+^ population, express at least two WC1 genes based on reactivity with two mAbs: mAb CACT21A that reacts with WC1-8 only and mAb BAG25A that reacts with WC1.1-like molecules but not WC1-8 (5, 24). However, the following questions remained: is there a specific number of different WC1 genes that all WC1^+^ γδ T cells express and are there set combinations of WC1 genes always expressed together? Understanding the expression of WC1 genes on individual γδ T cells is necessary to build models of how to induce cellular immune responses to specific pathogens. By analyzing WC1^+^ γδ T cell clones here, we showed that the WC1 locus is permissive for transcription of more than one gene by an individual cell and describe the many combinations of WC1 gene transcription. Finally, using the *Leptospira*-responsive WC1^+^ memory γδ T cell clones we showed these cells have a high propensity to express WC1 molecules that bind to the pathogen.

## Materials and Methods

### Isolation of Cells and RNA

Whole blood was collected with heparin by jugular venipuncture from a single adult Holstein heifer that had been vaccinated against *Leptospira* serovar Hardjo with a commercial inactivated vaccine (Spirovac, Pfizer) using an initial two-dose regime during calfhood and subsequently boosted intermittently with a single dose during adulthood as approved by the University of Massachusetts IACUC. Rabies vaccines were given. This vaccination procedure in cattle has been shown previously to result in γδ T cells in blood that respond in recall responses when stimulated *in vitro* with *Leptospira* (6, 7, 27). Peripheral blood mononuclear cells (PBMC) were isolated from blood by Ficoll-hypaque density gradient centrifugation. Total RNA was isolated from cells using TRIzol (Invitrogen) with 20 µg of glycogen carrier added after the chloroform step. 0.5–1 µg RNA was treated with 1 U of DNase enzyme (Promega) for 30 min at 37°C, then 70°C for 5 min. RNA purity and concentration were determined by Nanodrop spectrophotometry (Thermo-Fisher). cDNA synthesis was done using a commercial reverse-transcriptase kit (Promega) according to the manufacturer’s protocol. For T cell clone cDNA samples, we used Single Cell PreAmp Mix with random primers (Fisher Scientific), at 95°C for 10 min, followed by 14 cycles of 15 s at 95°C and 4 min at 60°C with a final inactivation at 99°C for 10 min.

### Lymphocyte Cultures

Lymphocytes were cultured with a modification of the protocol for generating bovine central memory T cells (T_CM_) (28). Briefly, 2.5 × 10^6^ PBMC were stimulated in a 24-well culture plates in 1 ml of complete RPMI (c-RPMI) medium [RPMI-1640 supplemented with 10% heat-inactivated fetal bovine serum (Hyclone), 200 mM l-glutamine (Sigma), 5 × 10^−5^ M 2-mercaptoethanol (Sigma) and 10 mg/ml gentamycin (Invitrogen)] with 0.16 µg/ml of sonicated *Leptospira borgpetersenii* serovar Hardjobovis. On days 3 and 7, 0.5 ml of supernatant was removed from each well and replenished with 0.5 ml of c-RPMI containing 30 U/ml of recombinant bovine IL-2 (rBoIL-2; R&D Systems). On days 10 and 12, 0.5 ml of the supernatant was removed and replaced with fresh c-RPMI medium. This protocol is referred to as the T_CM_ protocol throughout. On day 14, cultures were pooled and washed with sterile PBS and then dye-loaded with 0.5 µM efluor-670 cell division dye (eBioSciences) according to the manufacturer’s protocol. Cells were cultured with sonicated *Leptospira* for an additional 7 days; this modification is referred to as the T_EM_ protocol.

### Immunofluorescence Staining and Flow Cytometric Sorting

WC1.1^+^ and WC1.2^+^ γδ T cell subpopulations were obtained by staining lymphocytes with anti-WC1.1 mAb (BAG25A with anti-Mu IgM-FITC) and anti-WC1.2 mAb (CACTB32A with anti-Mu IgG1-PE). For WC1.3^+^ cells, lymphocytes were stained with anti-WC1.1 mAb and anti-WC1.3 mAb (CACT21A with anti-Mu IgG1-PE). Double or single-stained cells were sorted using FACS ARIA (BD). To obtain cell populations for T cell expansion, efluor670 dye-loaded cells were washed and stained as above, but sorted on efluor670-APC low cells (indicating multiple cell divisions had occurred in culture) to obtain WC1.1, WC1.2, and WC1.3 cell populations. Evaluation of memory markers by indirect immunofluorescence with mAbs against TCRδ (mAb GB21A, IgG2b), CD44 (mAb BAQ40A, IgG3-PE), and CD62L (mAb BAQ92A, IgG1-PE) and appropriate secondary Abs conjugated to fluorophores was conducted and evaluated by flow cytometry. MAbs were purchased from Washington State University Monoclonal Antibody Center (Pullman, WA, USA), and fluorophore-conjugated secondary polyclonal antibodies were purchased from Southern Biotechnology. All analyses were done using FlowJo v10 (TreeStar, Inc.).^1^

### Generation of T Cell Clones

On day 21 of the T_EM_ protocol, sorted WC1^+^ γδ T cell populations were plated into 96-well round-bottom tissue culture plates at a concentration of 1 cell/well in 100 µl volume. Plated cells were stimulated by adding 5 × 10^4^ irradiated (5,000 rads) autologous PBMC and 10 U/ml rBoIL-2 with or without 0.5 ng/ml rHuIL-15 (R&D Systems) with or without 0.16 µg/ml sonicated *Leptospira borgpetersenii* serovar Hardjbovis. Every 10 days, 100 µl of supernatant from each well was removed and replaced with 100 µl medium containing the same components until cells were harvested. Cell colonies were visible by 20 days after plating in 96-well plates and were harvested in TRIzol for RNA isolation about 7 weeks post-plating. The likelihood that any of the cell colonies had arisen from a single cell was determined by the method of de St. Groth (29). For each putative T cell clone, we harvested between 5 × 10^4^ and 5 × 10^5^ cells for cDNA synthesis. Viable cells were counted in a hemocytometer by microscopy using Trypan Blue exclusion.

### Primer Design and qRT-PCR

ClustalW2^2^ was used for multiple sequence alignment for bovine WC1 SRCR a1 domains based on accession numbers as published (26) to design TaqMan assays for individual WC1 genes (Figure S1 in Supplementary Material). Custom designed primers and the FAM/MGB fluorophore/quencher system (referred to as TaqMan assays) were prepared by Invitrogen. Additionally, commercially available TaqMan assays for the bovine TCRδ constant gene (TRDC), GAPDH, beta-2 microglobulin, CD4 and CD8α were obtained from Invitrogen (Table 1). GAPDH and beta-2 microglobulin were used as housekeeping genes to determine template concentrations while CD4 and CD8α were used as negative controls (data not shown). Amplicons were generated using the Stratagene qPCR system with the following settings: an initial 2 min UDG step followed by 10 min at 95°C and 40 cycles of 15 s at 95°C, and 30 s at 60°C. The mean and SEM of replicate wells for WC1 and TRDC TaqMan assays was calculated for each target.

**Table 1:** TaqMan primer/probe assays.

Gene ID	Genbank accession #	TaqMan assay ID	Corresponding plasmid for producing standard curves
*wc1-1*	FJ031186	AIRSAUH	pSecTag2A-WC1-1
*wc1-2*	JN998897	AIS080P	pSecTag2A-WC1-2
*wc1-3*	FJ031191	AIT966X	pSecTag2A-WC1-3
*wc1-4*	FJ031202	AIVI5C5	pSecTag2A-WC1-4
*wc1-5*	JQ900627	AIWR3JD	pSecTag2A-WC1-5
*wc1-6*	JN234380	AIX01PL	pSecTag2A-WC1-6
*wc1-7*	JN234377	AIY9ZVT	pSecTag2A-WC1-7
*wc1-8*	JN998896	AI0IX11	pSecTag2A-WC1-8
*wc1-9*	FJ031208	AI1RV79	pSecTag2A-WC1-9
*wc1-10*	JQ900628	AI20UEH	pSecTag2A-WC1-10
*wc1-11*	FJ031209	AI39SKP	pSecTag2A-WC1-11
*wc1-12*	JN234378	AI5IQQX	pSecTag2A-WC1-12
*wc1-13*	FJ031187	AI6ROW5	pSecTag2A-WC1-13
*trdc*	D90419	AIGJR3Q	pCR2.1-TRDC
*gapdh*	NM_001034034	Bt03210913	pCR2.1-GAPDH
*cd4*	NM_001103225	Hs01058407	n.d.
*cd8*	NM_174015	Bt03212361	n.d.

*n.d., not done*.

Amplicons were run on 2% TAE-agarose gels, purified by gel extraction (Qiagen) (Figure S2A in Supplementary Material), cloned into pCR2.1 vector using TOPO-TA (Invitrogen), transformed into ONE-SHOT competent cells (Invitrogen), and plated on LB-kanamycin plates. Bacterial colonies were selected and the plasmids purified using the mini-prep plasmid purification kit (Qiagen). Selected cDNA clones were commercially sequenced by Genewiz (South Plainfield, NJ, USA) and results were analyzed using BioEdit (Version 7.0.5.3) and sequences aligned using Clustalw2 (see text footnote 2) (Figure S2B in Supplementary Material). The primers occasionally amplified a secondary SRCR a1 domain as well as the targeted one, determined by cloning and sequencing the amplicons. We tested for and found no off-target detection with the TaqMan assays (Table 2) when using previously generated constructs of WC1 SRCR a1-domain-coding sequences cloned into the pSecTag2 vector (23) as templates (WC1-pST2A). Thus, we interpreted this to mean that the FAM probe corrects any spurious transcript amplification by the primers. For generating standard curves, WC1-pST2A plasmids were diluted to equal concentrations by spectrophotometric quantification; primers to the vector backbone and qPCR were used to confirm the concentration of the constructs. TRDC amplified from PBMC was cloned into pCR2.1 and used to generate a standard curve.

**Table 2:** Evaluation of WC1 TaqMan assays for specificity.

TaqMan assays for	WC1 a1 domain in the pST2a vector as templates (WC1-x)												
	1	2	3	4	5	6	7	8	9	10	11	12	13
WC1-1	31.90^a^												
WC1-2		32.15											
WC1-3			30.94										
WC1-4				32.34									
WC1-5					29.97								
WC1-6						29.07							
WC1-7							30.02						
WC1-8								33.13					
WC1-9									26.87				
WC1-10										28.16			
WC1-11											30.05		
WC1-12												26.33	
WC1-13													29.28

*^a^Each number is a Ct value with blank cells meaning no Ct detected during amplification*.

### Statistics

Pearson’s *r* correlation was used to determine the relationship between the number of WC1 genes transcribed and the moles of either TRDC or WC1 gene transcripts measured. Student’s *t*-test was used to determine significant differences in the proportion of cells in the WC1 subpopulations with transcripts for WC1 genes whose products bind *Leptospira*.

## Results

### Derivation and Analysis of WC1^+^ γδ T Cell Lines and Clones

**Figure 1:**
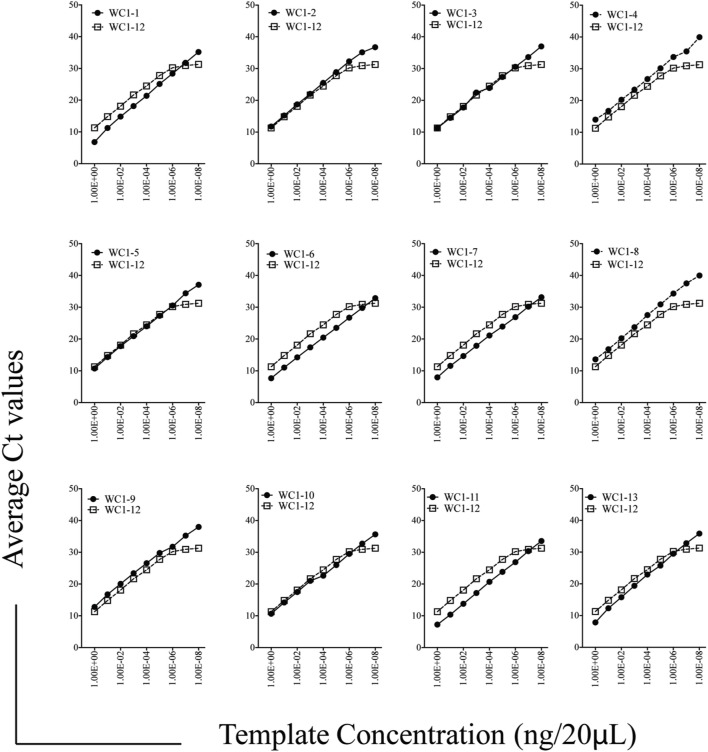
Efficiency of Taqman assays for WC1 transcripts. Standard curves for individual WC1 genes’ scavenger receptor cysteine rich a1 domains were amplified and detected with the Taqman primer/probe assays using serially diluted pST2a-WC1 templates. In each case, *WC1-12* efficiency is shown as a benchmark.

**Figure 2:**
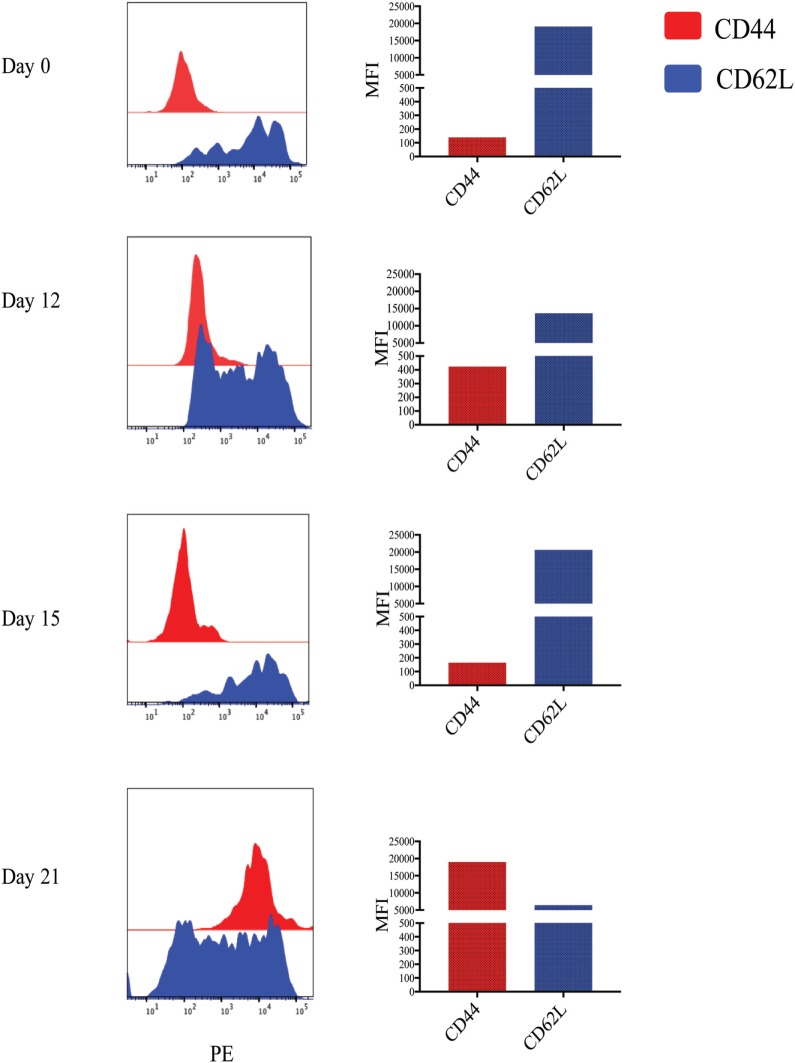
Memory markers of *Leptospira* stimulated cultures. Cultures of peripheral blood mononuclear cells from a vaccinated animal subjected to the T_CM_ and T_EM_ protocol and cells stained for T cell receptor (TCR)δ with monoclonal antibody (mAb) GB21A (FITC) at the times indicated. The TCRδ^+^ cells were flow cytometrically gated on mAb GB21A^+^ and evaluated for their surface marker expression of either CD44 or CD62L (PE) by two-color immunofluorescence shown as histograms. The mean fluorescence intensity (MFI) is shown in the bar graphs.

While previous studies indicated that bulk γδ T cell subpopulations can express more than one WC1 gene (9, 24), expression of WC1 genes by individual γδ T cells has been largely undefined. To fill this gap, we evaluated WC1 gene transcription by clones of WC1^+^ γδ T cells using a customized TaqMan assay system. Due to a high degree of sequence similarity among WC1 genes (Figure S1 in Supplementary Material), a number of experiments were performed to validate the specificity of the TaqMan assays (see M&M: Table 2; Figure S2 in Supplementary Material) and differences in efficiency (*Ct* values using the plasmids with cloned WC1 SRCR a1 domains ranged from *Ct* 26 to 33 when equal concentrations of template were used) were compensated for by constructing standard curves (Figure 1); the difference in efficiency was accepted as a necessary compromise to maintain the ability of the TaqMan assays to distinguish among WC1 transcripts. Bulk cell lines were generated from which to derive γδ T cell clones using a mixture of *Leptospira* and cytokine cocktail stimulations. γδ T cells in PBMC from *Leptospira*-vaccinated animals but not unvaccinated animals (6) are known from our previous studies to have recall responses *in vitro* (27). We evaluated the γδ T cells in the expansion cultures with *Leptospira* for changes in expression of memory markers as we have shown previously these differ from the expression by naïve cells from unvaccinated cattle (27). An increase in CD44 and decrease in CD62L was seen in the initial round of stimulation using a protocol to generate bovine T_CM_ cells (28) (Figure 2, day 0–day 12). There was an even greater increase in CD44 expression and decrease in CD62L by day 21 (Figure 2) when the dividing cells were sorted for T cell cloning (a time when the cells would be ready to respond to a new round of stimulation). Variations in the cloning protocol after day 14 were tried with a total of four distinct strategies and included sorting populations of dividing cells to enhance the chance of clonal diversity (Table S1 in Supplementary Material). Clones were generated from 12 cycles of expansion (Table S1 in Supplementary Material) with purity of all the flow cytometry sorts ranging from 87.9 to 99.3% (Figure S3 and Table S1 in Supplementary Material). WC1.1^+^ and WC1.1^+^/WC1.3^+^ sorted cells were cultured with IL-2 and *Leptospira* (Table S1 in Supplementary Material, Strategy 3) since we knew that some WC1.1^+^ (7) and WC1.3^+^ cells (Chen C and Baldwin CL, unpublished data) from vaccinated animals are *Leptospira* responsive. Clones from these sorted populations that were expanded by re-stimulation with *Leptospira* grew more efficiently than those without. To obtain WC1.2^+^ clones IL-2 alone or in combination with IL-15 with or without IL-18 was used, since IL-15 is known to stimulate γδ T cells (30, 31). In total, we generated 78 clones (Table 3). The cloning efficiency of each cycle showed that the probability that any individual T cell line was actually a clonal population was 96–99% based on the maximum likelihood method of de St Groth [Table S2 in Supplementary Material; (29)].

**Table 3:** Summary of WC1 transcripts expressed by γδ T cell clones.

Clone #	# of WC1 genes transcribed	WC1.2-like WC1 transcripts (WC1-)	WC1.1-like WC1 transcripts (WC1-)
A86	1	4	–
23		4	–
13M-20-2D		7	–
A6C		7	–
101F		7	–
8D2		7	–
3-E11		7	–
PY08C		–	1
2D10-21A		–	1
3-10H		–	3
5-11G		–	3
131-6-11B		–	6
B11D		–	10
6-7C		–	10
28-9H		–	12
A93		–	13
AC06		–	13
BG08		–	13
5	2	4, 7	–
33		4, 7	–
17		4, 7	–
AE05		4, 7	–
159E		4, 7	–
3-7G		–	3, 8
2-7C		–	3, 8
8I		–	1, 11
501K/D		–	6, 13
25-1F		4	8
8G-31		4	10
2		4	11
26-3F-13N		4	11
7H5		4	11
87-30		4	13
7		7	6
5-11F		7	8
21		7	1
209E		9	10
1-3H	3	–	1, 8, 11
13L-10-5E		–	1, 11, 12
17-10A		–	2, 12, 13
B7B		–	8, 10, 11
18-10F		–	3, 8, 13
PY01G		4, 7	1
4-E6		4, 7	6
B9D		4, 7	11
10		4, 7	11
16		4, 7	11
4-E8		4, 7	11
21-3E		4, 7	12
9		4	7, 11
8E-29		4	6, 11
23-9H-13E		7	8, 11
7-10A-13C		7	8, 11
AF08		7	11, 13
8M		9	1, 11
101G		9	3, 10
24		9	6, 10
8P	4	–	3, 6, 10, 11
9H11		4	3, 6, 10
B8F		4	1, 8, 11
20-7H		7	3, 11, 12
6		4,7	1, 11
13		4,7	9,11
7H5		4, 7	1, 3
2D10		4, 7	1, 3
22-9H		4, 7	3, 12
27-5F		4, 7	3, 12
6-9D-13B		4, 7	8, 11
13K-13-9B		4, 7	11, 13
3G6		4, 9	11, 12
PYO1E		7, 9	1, 11
1B5-21A	5	7	1, 6, 11, 13
PY100		4, 7	1, 6, 8
4		4, 7	8, 11, 13
11-9F		4, 9	1, 8, 11
A5F	6	4	3, 8, 11, 12, 13
9H10		4, 7	1, 3, 6, 10
20-9G		4, 7	2, 10, 11, 12

### Variegated WC1 Gene Transcription by γδ T Cell Clones

**Figure 3:**
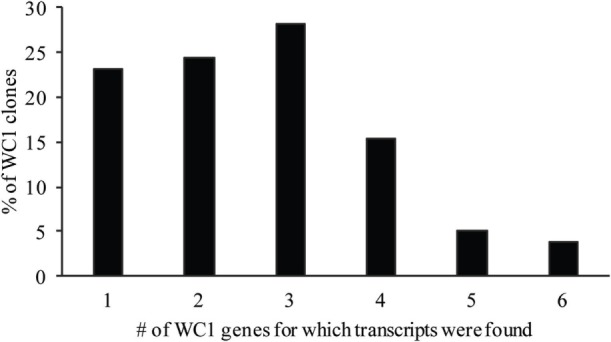
Percentage of WC1 transcripts expressed per γδ T cell clone. The percentage of clones with a particular number of different WC1 gene transcripts is shown.

**Figure 4:**
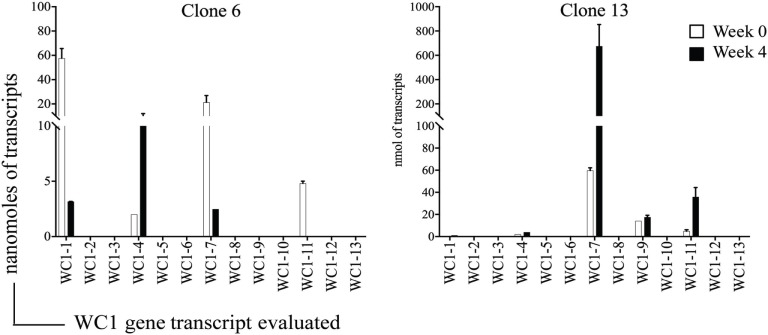
Time-course evaluation of WC1 gene transcription. Evaluation of WC1 gene transcription by selected clones at two different time-points 4 weeks apart are shown. Filled bars show earlier time point and open bars the later. Transcripts are represented as moles of the mean ± SE of replicates. This was done three times with triplicate samples.

**Figure 5:**
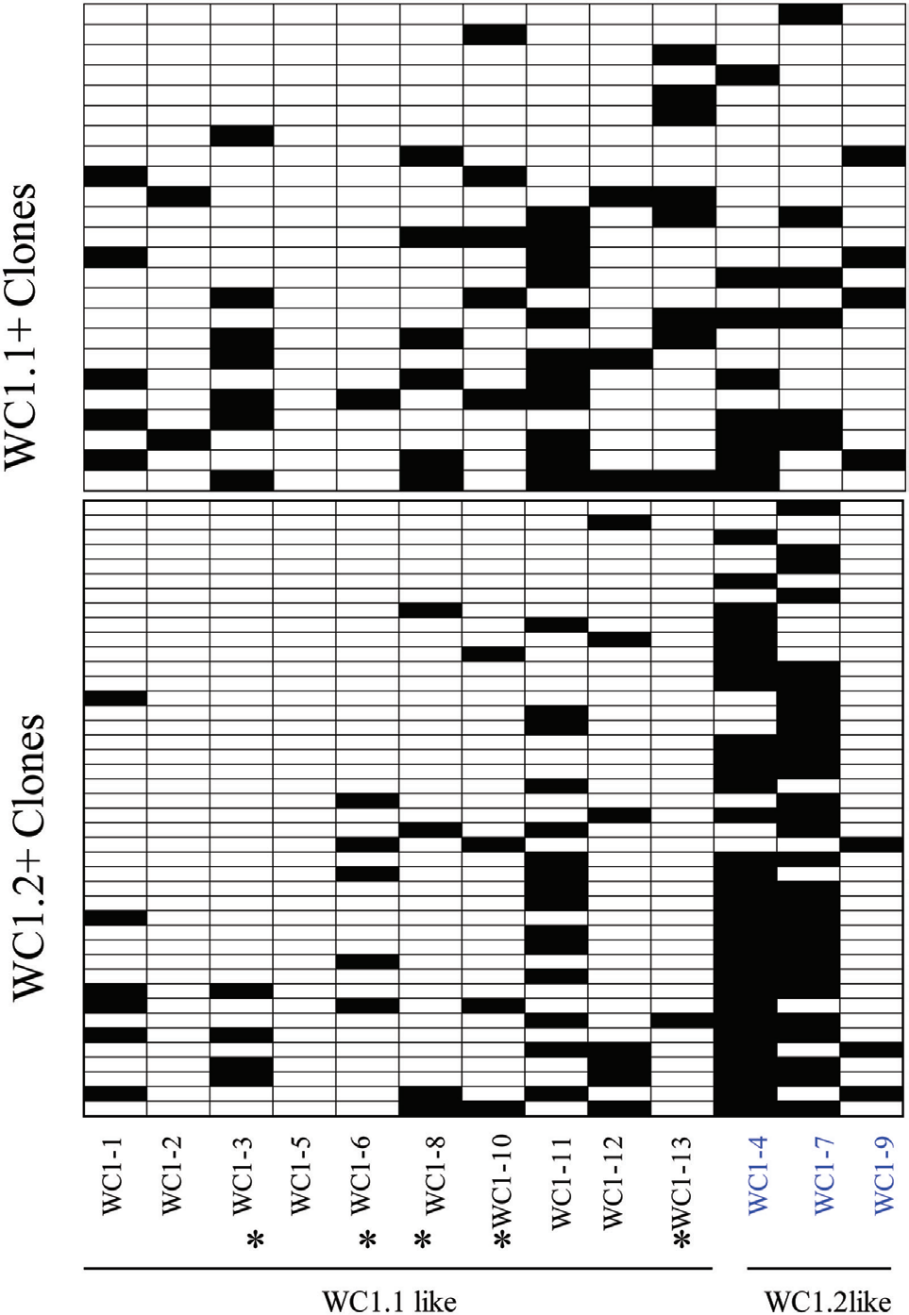
WC1 gene transcripts associated with individual WC1.1^+^ or WC1.2^+^ clones. The various combinations of WC1 gene transcripts are shown for clones derived by flow cytometrically sorted cell subpopulations in a heat map. * = WC1 gene products that bind *Leptospira*.

Analysis of the gene transcription by the γδ T cell clones showed the number of WC1 genes transcribed per clone varied and that many more combinations occurred than expected (Table 3). If the mean and SE was at zero, the gene was not included in the tally of transcription since this indicated it was not measurable in all replicate samples. T cell clones expressing only 1–3 WC1 genes accounted for ~75% of all γδ T cell clones (Figure 3) with 8 of the 13 WC1 genes found to be transcribed alone in individual clones. The consistency of transcription over time for sample clones was evaluated (Figure 4) and showed that while the magnitude of transcription was not identical (e.g., see Clone 6: at Wk 0 WC1-1 has the greatest level of transcription while at Wk4 WC1-4 did) the transcription of specific genes was generally consistent. While cDNA preparations from clones had different molar concentrations of transcripts for WC1 genes and TRDC (shown for representative clones in Figure S4 in Supplementary Material), neither correlated with the number of different WC1 gene transcripts detected (Pearson’s *r* correlations were 0.01662 and 0.00016, respectively). While evaluation of all putative clones showed that the frequency that had transcripts from five or six different WC1 genes was low (11% of the colonies) (Figure 5), based on the prediction that only 1–4% of the colonies that arose are not clonal populations using the maximum likelihood method (29) then at least some of the colonies transcribing five or six different WC1 genes would be expected to have been derived from a single cell.

**Figure 6:**
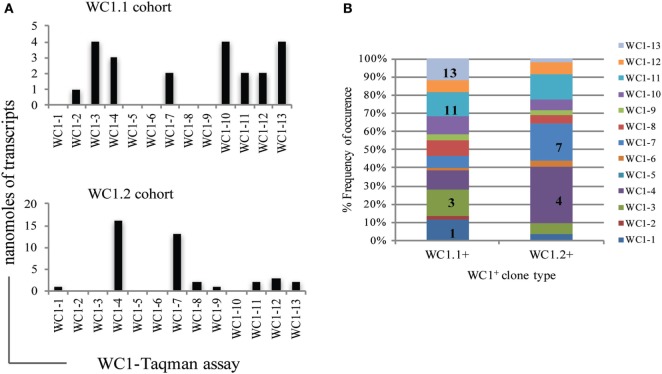
Diversity index of individual WC1 gene transcription by γδ T cell clones in each WC1 cohort. **(A)** For each clone the predominately expressed WC1 gene, based on highest mean transcript level, was noted and the total number of times a WC1 gene was the predominant is plotted. **(B)** Diversity index shows the percentage of times transcripts for the WC1 genes occurred in a cohort of clones (*y*-axis). WC1 genes most frequently transcribed within the repertoire of WC1 transcripts is indicated as a number *x* (e.g., WC1-*x*) in appropriate boxes to add emphasis.

Previous analyses of bulk-sorted WC1^+^ subpopulations indicated that the WC1 genes with the highest levels of transcription differed between the WC1.1 and WC1.2 subpopulations (9, 24). Here, we found the transcription of WC1 genes overlapped among clones from the two subpopulations (Figure 5) but when just the gene with the highest WC1 transcript level for each T cell clone was considered and re-aggregated (Figure 6A) the pattern of relative transcription was generally similar to what we reported for bulk-sorted subpopulations previously. The diversity index also showed that there are differences in WC1 gene transcription between WC1.1 and WC1.2 clones (Figure 6B). Nevertheless, unexpected combinations of transcripts were found together, although the archetypal genes for the WC1.1 and WC1.2 subpopulations (*WC1-3* and *WC1-4*, respectively) were only detected together in cell lines that had transcripts for four or more WC1 genes (Table 3). The infrequent occurrence of this phenomenon considering all 78 clones evaluated largely supports our previous findings that WC1.1 and WC1.2 are the two foundational sub-lineages of WC1^+^ γδ T cells expressing an array of WC1 genes that enable them to respond to specific pathogens differently.

### WC1 Gene Transcript by Pathogen-Specific Memory γδ T Cells

A proportion of the cells in the WC1.1^+^ subpopulation of γδ T cells from animals that have been primed by vaccination specifically proliferate and produce IFNγ in response to stimulation with *Leptospira* in recall assays indicating their role as adaptive-like memory lymphocytes (27). In contrast, many fewer in the WC1.2^+^ subpopulation do so. Based on these observations, we sought to determine if the WC1 gene transcription was different among clones that responded to *Leptospira* (WC1.1 cohort) compared with those that generally did not (WC1.2 cohort) since we have shown previously that 5 of the 13 bovine WC1 molecules have SRCR domains that directly bind *Leptospira* (i.e., WC1-3, WC1-6, WC1-8, WC1-10, and WC1-13) (23). Since antibody-mediated co-ligation of WC1 and TCR/CD3 results in augmented proliferation and IFNγ production, it suggests that pathogen-mediated co-ligation of WC1 and TCR/CD3 would also and thus that WC1 expression and pathogen binding specificity would influence γδ T cell responsiveness (24, 25, 32). 80% of the WC1.1 cohort of clones that were generated with cells from a primed animal and continually cultured with *Leptospira* had transcripts for at least one WC1 gene that had a binding domain for *Leptospira* (Figure 5). Clones from the WC1.2 cohort were necessarily developed with cytokine stimulation here and only 37% of those clones had transcripts coding for WC1 molecules with a binding domain for *Leptospira*. This was significantly different (*p* < 0.008). The observation that some WC1.2^+^ cells do have transcripts for *Leptospira*-binding WC1 molecules is consistent with the minor population of WC1.2^+^ γδ T cells that have been found to respond to *Leptospira* stimulation (7) and that would be preferentially expanded under these conditions given the initial culture was with *Leptospira*.

## Discussion

We previously hypothesized that within the WC1.1^+^ and WC1.2^+^ subpopulations there would be set WC1 gene programming that broke down these cohorts into even smaller populations. This was rejected based on the results here because few clones had the same pattern of WC1 gene transcription. This mix of patterns occurred even though all the clones were derived from a single animal. We would expect similar complex patterns in other animals since unlike the gene numbers for Ly49 and KIR (other multigenic arrays of immune system cell receptors) that are highly variable among the genomes of mouse strains (33) and humans (34), respectively, the WC1 family is highly conserved with regard to gene number and sequence among cattle breeds including for *Bos taurus* and *Bos indicus* (3, 26). Although the gene transcription and presumably expression of the WC1 multigenic array was more complex and variable than predicted it is similar to the 30 different Ly49 patterns seen for 62 clones of murine NK cells (35) and agrees with TLR gene expression by human and murine CD4 and CD8 T cells which ranges from four to seven TLR [reviewed in Ref. (36)] and with transcription of one to eight KIR family genes by human NK clones (37). However, for NK cells the number of different KIR genes transcribed/cell is normally distributed (37) while our results indicated transcription of fewer WC1 genes per cell is favored.

Variegated gene expression of immune cell receptors that sense the environment, including KIR, Ly49, and TLR, is common and allows individual cells to detect specific stimuli (38). Some multigenic arrays of PRR such as TLR have some genes constitutively expressed on lymphocytes while expression of other genes is induced in response to environmental cues such as microbes and cytokines (39). In contrast, the WC1 transcription patterns appear to be stable since among animals there is a largely consistent ratio of WC1.1^+^ and WC1.2^+^ cells, defined by their WC1 gene expression, that changes according to age (7) and which first appears during thymic development (Damani-Yokota, unpublished data). This is similar to the set proportion of cells expressing specific KIR genes in human or Ly49 genes in mice (40–42). Variegated gene expression of these multigenic arrays has been shown to be controlled by differential methylation of CpG islands around the transcriptional start site (43), by bidirectional promoter switches that are controlled by noncoding RNA (38, 42, 44), and by differences in 5′ upstream regulatory regions (41) which ensure stability of gene expression. Finally, there is precedent for the SRCR receptor transcriptome in a population of immune cells changing in response to bacterial injection into sea urchins (45), suggesting that the clonal expansion of cells with stable expression of SRCR receptors in response to infection is an ancient and conserved mechanism.

The WC1 transcriptome of the two major subpopulations of WC1^+^ γδ T cells (WC1.1^+^ and WC1.2^+^) isolated from either *ex vivo* PBMC or following culture with antigens have signature WC1 genes dominantly transcribed but they also have lower levels of transcripts for additional WC1 genes (9, 24). These minor transcripts could represent low-level sterile transcription since their gene products were not evident by immunofluorescence. For example, sterile transcripts for TLR are found in CD4 T cells (46). Nevertheless, some of the clones had transcripts that coded for WC1 genes normally expressed by the other cohort that may have arisen from impurities in the flow cytometric sorting process (i.e., WC1.1^+^ cells in the WC1.2^+^ sorted population) while others may be cell lines, i.e., not of single cell origin. The presence of two or more clonal populations of cells in a culture could convey a growth advantage to cells including some not activated directly by antigen. These issues are unresolved but would account for only a small percentage of the colonies or cell lines derived since the maximum likelihood method (29) indicates that there is 96–99% probability that they are derived from a single cell.

**Figure 7:**
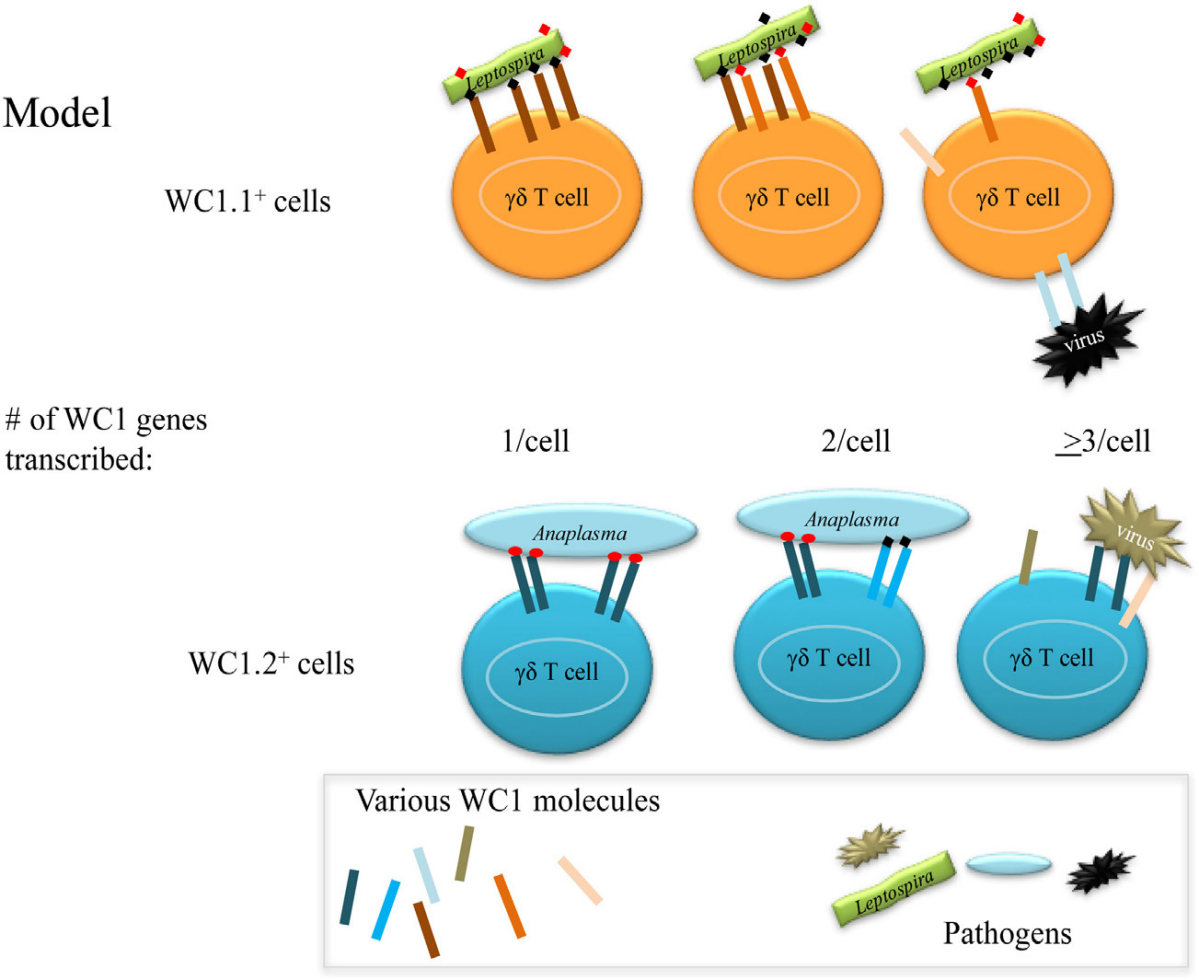
Model for WC1 interaction with pathogens. γδ T cells may express variable numbers of different WC1 molecules thus potentially enabling them to interact with more than one ligand on the same or different pathogens. However, since the number of WC1 co-receptors is necessarily limited on a cell an increase in receptor diversity could affect the avidity of the interaction with any particular ligand.

Our previous studies support a model of γδ T cell activation in which pathogen ligation of the WC1 co-receptors along with the TCR is necessary for optimal cellular responses (32, 47). Moreover, crosslinking of the γδ TCR with WC1s using an mAb that recognizes most WC1 molecules (mAb CC15) has been shown to give a stronger and more amplified signal compared with crosslinking with an mAb that recognizes a specific WC1 molecule within the family of 13 (i.e., WC1-8 by mAb CACT21A) (24). Thus, co-crosslinking of all WC1 molecules on a cell with the γδ TCR by natural ligand binding would be expected to provide an advantage to the cell while multiple different WC1 molecules increase the potential for WC1 molecules to bind more than one ligand of a pathogen. Also expression of WC1 molecules in various combinations could increase the types of pathogens to which an individual cell could respond. Conversely, expression of multiple WC1 genes could decrease avidity for a particular pathogen if some of the WC1 molecules did not bind a ligand on the particular pathogen. We present these alternatives in a model (Figure 7).

Since much of our work has used the recall response to *Leptospira* as the model to elucidate the role of WC1 molecules in directing γδ T cell responses, we framed the results in that context. Our hypothesis that the individual or combination of WC1 molecules being expressed by a γδ T cell contributes to its pathogen responsiveness is supported by our finding that the majority of memory γδ WC1.1^+^ T cell clones had transcripts for WC1 molecules that bind *Leptospira* (23). Since we were unable to grow WC1.1^+^ clones without repeated stimulation with *Leptospira*, we suggest that the remaining clones that did not express WC1 molecules with known *Leptospira*-binding domains may have a TCR with higher affinity for the bacteria and thus do not need co-ligation of WC1 for activation. With regard to the WC1.2^+^ population, it is known to be characterized by dominant transcription of genes *WC1-4, WC1-7*, and *WC1-9*, none of which have been found to bind *Leptospira* (23). However, some WC1.2^+^ γδ T cell clones had transcripts for WC1 genes whose products bind *Leptospira* which agrees with the small proportion of WC1.2^+^ γδ T cells in PBMC that do in fact respond to *Leptospira* (7).

The cloning system yielded valuable insights into the variegated WC1 gene transcription but the question of how stable WC1 transcriptional programming is established in thymocytes remains unresolved. We are currently investigating differential occupancy of WC1 enhancers in the WC1.1 and WC1.2 loci by transcription factors implicated in γδ T cell development (12) and consequent epigenetic modification of the loci to help resolve this question. It also will be important to characterize the TCR repertoires of those *Leptospira*-responsive WC1.1^+^ γδ T cell clones that did not have transcripts for *Leptospira*-binding WC1 molecules. We predict that the CDR3 repertoires for these cells will be significantly different from those with *Leptospira*-binding WC1 co-receptors and are the subject of current studies.

## Ethics Statement

All animal use was approved by the University of Massachusetts’ Institutional Animal Care and Use Committee.

## Author Contributions

PD-Y contributed to the design of experiments, acquisition, analysis, and interpretation of the data and helped draft the manuscript. CB and JT contributed to the conception and design of the research, analysis and interpretation of the data, and drafting the manuscript. All authors critically revised, read and approved the final manuscript, and agreed to be fully accountable for ensuring the integrity and accuracy of the work.

## Conflict of Interest Statement

The authors declare that the research was conducted in the absence of any commercial or financial relationships that could be construed as a potential conflict of interest.

## Footnotes

^1^https://www.flowjo.com (Accessed: December, 2017).

^2^http://www.ebi.ac.uk/Tools/clustalw2/index.html (Accessed: December, 2017).

## Supplementary Material

Figure S1 Sequence alignment of bovine WC1 genes. General structure of bovine WC1 molecules is shown for 6 and 11 scavenger receptor cysteine rich (SRCR)-containing molecules along with the transmembrane (TM) and endodomain. Multiple sequence alignment of the deduced amino acids of the SRCR a1 domains (the most membrane distal) of the 13 bovine WC1 molecules using ClustalW with identical amino acids shown as dots and gaps as dashes. Variable regions 1 and 2 (VR1 and VR2, respectively) are marked to show regions of greatest sequence variability among the different WC1 sequences. WC1-4, WC1-7, and WC1-9 are considered WC1.2-type molecules while the rest are WC1.1 based on amino acid deletions or additions at positions 75, 76, and 89.

Figure S2 Establishment of TaqMan primer assays. **(A)** Evaluation of TaqMan primer amplified PCR products on 2% TAE-agarose gel showing amplicon size ranging from 100 to 200 bp for WC1 transcripts labeled WC1-1 to WC1-13 and other genes as indicated. **(B)** PCR products were gel-purified and cloned into pCR2.1 and subsequently analyzed with Sanger sequencing. Multiple sequence alignment using BioEdit shows nucleotide sequences of TaqMan assay-amplified WC1 genes from cDNA relative to the reference gene sequence found in Genbank (see Table 1 for accession numbers).

Figure S3 Sorting strategy to obtain WC1^+^ γδ T cell subpopulations for single cell cloning. **(A)** Single-positive WC1.1^+^ or WC1.2^+^ and **(B)** double positive WC1.1^+^/WC1.3^+^ γδ T cells were flow cytometrically analyzed and gates applied. The three gated cell populations were then evaluated for their level of cell division dye and the efluor-670^low^ cells (indicative of multiple cell divisions) were collected as shown. This is representative of multiple flow cytometric sorts.

Figure S4 Representative clones with variable numbers of WC1 gene transcripts. Examples (from the 78 total clones) that had transcripts for one to five WC1 gene transcripts. If the mean was less than 2 and SE was at below zero, the gene was not included in the tally of transcripts in Figures 5 and 6 or Table 3. **(A)** WC1.1 cohort of γδ T cell clones from monoclonal antibodies (mAb) BAG25A^+^/CACTB32A^−^ sorted cells expanded using expansion strategy 3 (*Leptospira* and IL-2) or **(B)** WC1.2 cohort of γδ T cell clones from mAb BAG25A^−^/CACTB32A^+^ sorted cells expanded with IL-2 with or without IL-15 and IL-18 supplementation. Moles of transcripts for each clone (mean ± SE) for WC1 and TRDC (hatched bars) are shown.
