# Supplementary material for: Variegated Transcription of the WC1 Hybrid PRR/Co-Receptor Genes by Individual γδ T Cells and Correlation With Pathogen Responsiveness
Source: Front Immunol. 2018 May 7;9:717. doi: 10.3389/fimmu.2018.00717 (PMC5949365; doi:10.3389/fimmu.2018.00717)
Supplement: Supplementary file 1 [file Table_1.DOCX]

Table S1. T cell expansion strategies used to generate γδ T cell clones.

| T cell expansion  strategy |  | Day 14 | | | Day 21: Cells sorted to obtain these populations | | | Additives to cultures when plated by limiting dilution | | | Additives for re-stimulation on days  31, 41, 51 and 61 | |
| --- | --- | --- | --- | --- | --- | --- | --- | --- | --- | --- | --- | --- |
|  | Cycle # | Efluor-  670^a^ | Ag^b^ | IL-2 | WC1.1+  /WC1.2- | WC1.2+/  WC1.1- | WC1.1+/  WC1.3+ | Ag^b^ | IL-2 | Il-15 | IL-2 | IL-15 + IL-18 |
| Master |  | ♦ | ♦ | ♦ |  |  |  |  | ♦ |  | ♦ |  |
| S1 | I | nd | ♦ | ♦ |  |  |  | ♦ | ♦ |  | ♦ |  |
| S1 | II | nd | ♦ | ♦ |  |  |  |  | ♦ |  | ♦ |  |
| S2 | V | ♦ | ♦ | ♦ | ♦ |  |  |  | ♦ |  | ♦ |  |
| S2 | V | ♦ | ♦ | ♦ |  | ♦ |  |  | ♦ | ♦ | ♦ |  |
| S3 | III, IV, VI, IX, XII | ♦ | ♦ | ♦ | ♦ |  |  | ♦ | ♦ |  | ♦ |  |
| S3 | VII, X, XII | ♦ | ♦ | ♦ | ♦ |  | ♦ | ♦ | ♦ |  | ♦ |  |
| S4 | XII | ♦ | ♦ | ♦ |  | ♦ |  |  | ♦ |  | ♦ | ♦ |
| S4 | VIII | ♦ | ♦ | ♦ |  | ♦ |  |  | ♦ |  | ♦ | ♦ |

1. Efluor-670 refers to cell-division dye.
2. Ag refers to sonicated *Leptospira*.
