# Supplementary material for: Variegated Transcription of the WC1 Hybrid PRR/Co-Receptor Genes by Individual γδ T Cells and Correlation With Pathogen Responsiveness
Source: Front Immunol. 2018 May 7;9:717. doi: 10.3389/fimmu.2018.00717 (PMC5949365; doi:10.3389/fimmu.2018.00717)
Supplement: Supplementary file 2 [file Table_2.DOCX]

Table S2. Evaluation of the probability of being a T cell clone based on Poisson distribution.

| Temporal Cycle # | Total # wells seeded  (Day 0) | Day 20 post-plating | | | # wells with viable cells on  Day 48 | % cell lines that survived Day 20 to Day 48 |
| --- | --- | --- | --- | --- | --- | --- |
|  |  | % wells with growth | % Probability of being a clone^a.^ | # wells with cells |  |  |
| I | 480 | 2.08 | 99 | 10 | 7 | 70.00 |
| II | 576 | 8.68 | 96 | 50 | 4 | 8.00 |
| III | 480 | 8.96 | 96 | 43 | 8 | 18.60 |
| IV | 1920 | 1.30 | 99 | 25 | 5 | 20.00 |
| V | 480 | 10.42 | 98 | 50 | 5 | 10.00 |
| VI | 384 | 5.21 | 99 | 20 | 4 | 20.00 |
| VII | 1920 | 1.56 | 99 | 30 | 6 | 20.00 |
| VIII | 1920 | 1.56 | 99 | 30 | 11 | 36.67 |
| IX | 960 | 3.54 | 97 | 34 | 11 | 32.35 |
| X | 480 | 2.08 | 99 | 10 | 4 | 40.00 |
| XI | 1920 | 2.60 | 99 | 50 | 8 | 16.00 |
| XII | 2880 | 2.01 | 99 | 58 | 19 | 32.76 |

a. Determined by method of de St. Groth [[17](#_ENREF_17)].
