# Supplementary figures and images for: Variegated Transcription of the WC1 Hybrid PRR/Co-Receptor Genes by Individual γδ T Cells and Correlation With Pathogen Responsiveness
Source: Front Immunol. 2018 May 7;9:717. doi: 10.3389/fimmu.2018.00717 (PMC5949365; doi:10.3389/fimmu.2018.00717)

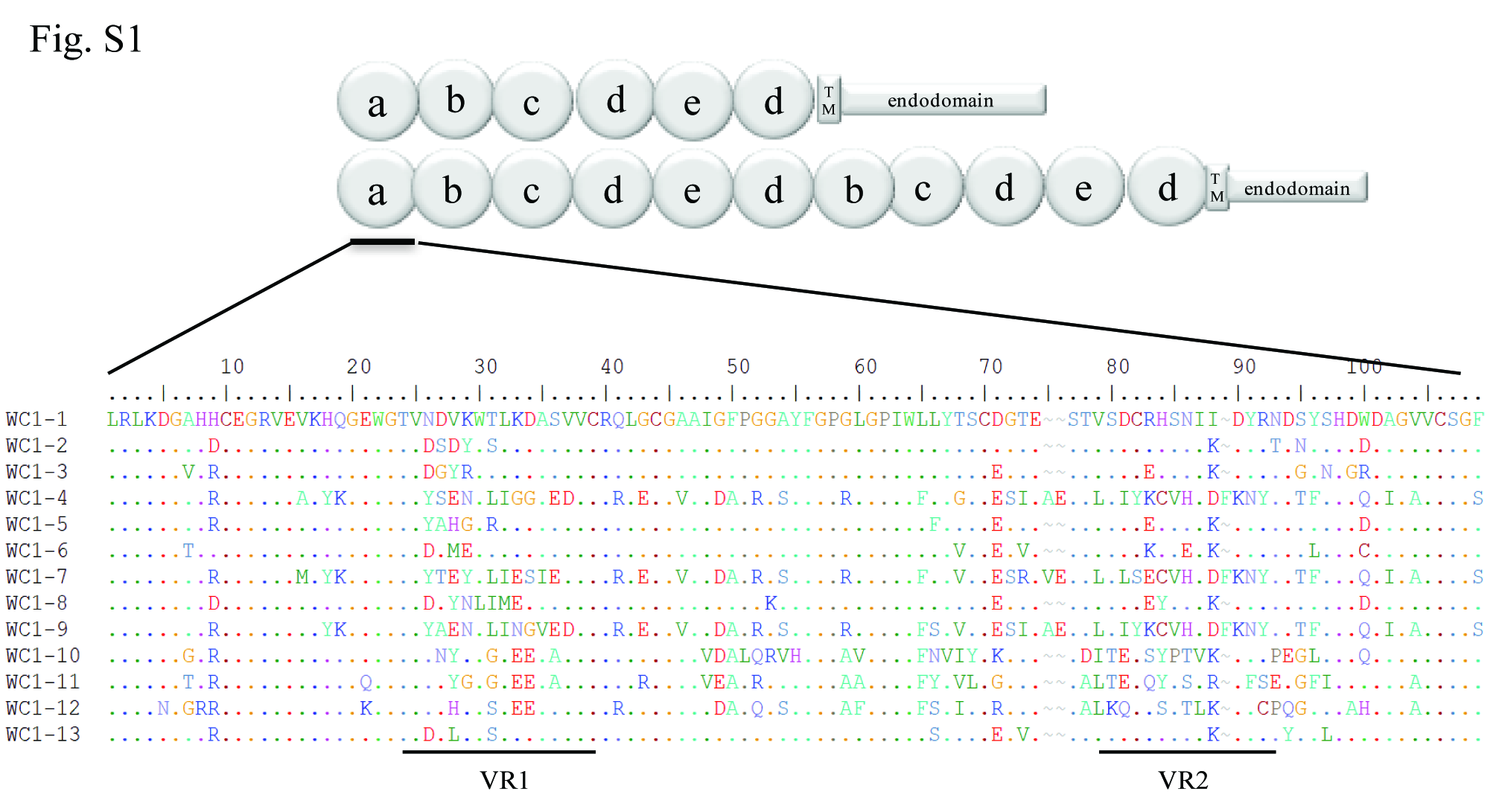

Supplement: Figure S1 — Sequence alignment of bovine WC1 genes. General structure of bovine WC1 molecules is shown for 6 and 11 scavenger receptor cysteine rich (SRCR)-containing molecules along with the transmembrane (TM) and endodomain. Multiple sequence alignment of the deduced amino acids of the SRCR a1 domains (the most membrane distal) of the 13 bovine WC1 molecules using ClustalW with identical amino acids shown as dots and gaps as dashes. Variable regions 1 and 2 (VR1 and VR2, respectively) are marked to show regions of greatest sequence variability among the different WC1 sequences. WC1-4, WC1-7, and WC1-9 are considered WC1.2-type molecules while the rest are WC1.1 based on amino acid deletions or additions at positions 75, 76, and 89. [file Image_1.TIF]

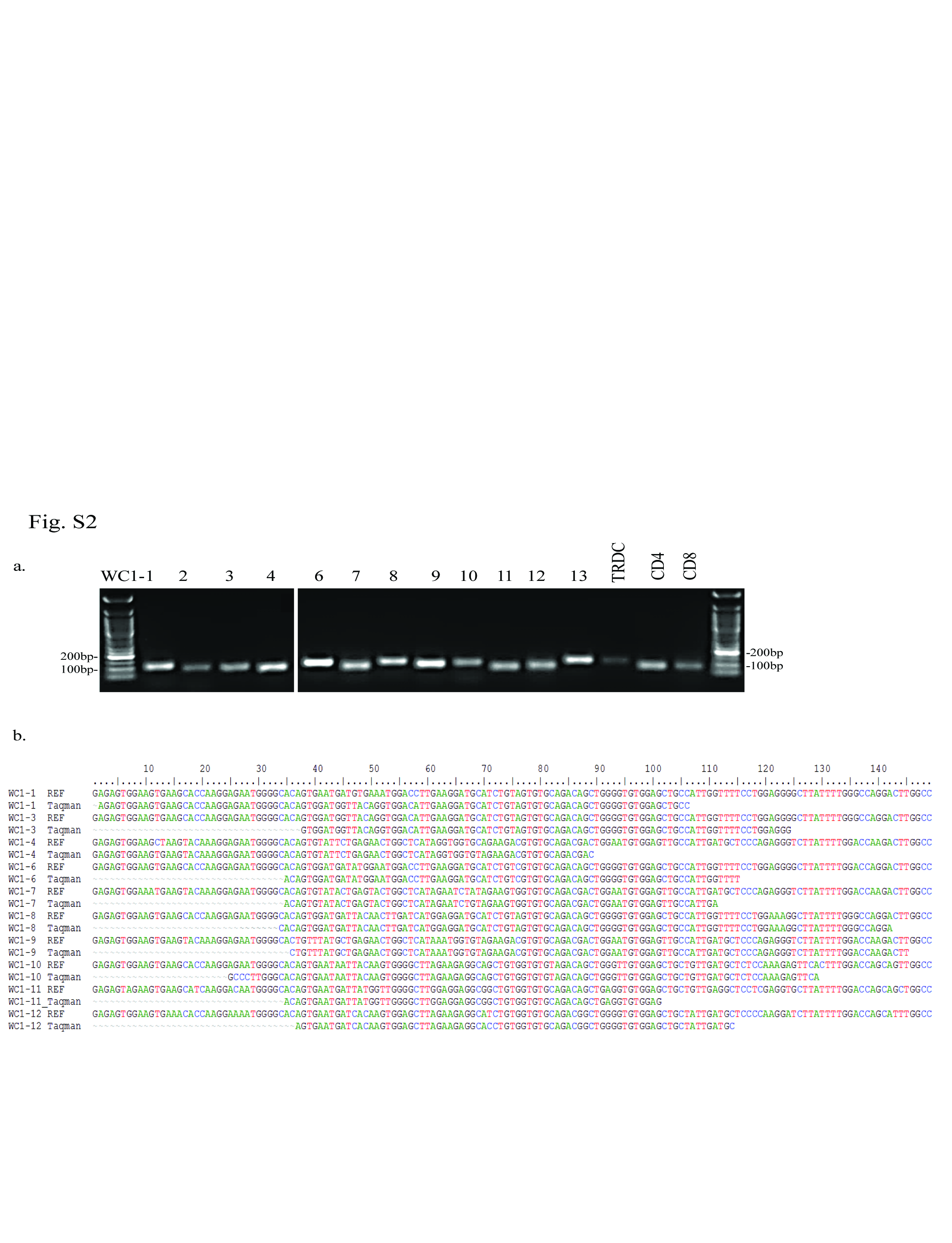

Supplement: Figure S2 — Establishment of TaqMan primer assays. (A) Evaluation of TaqMan primer amplified PCR products on 2% TAE-agarose gel showing amplicon size ranging from 100 to 200 bp for WC1 transcripts labeled WC1-1 to WC1-13 and other genes as indicated. (B) PCR products were gel-purified and cloned into pCR2.1 and subsequently analyzed with Sanger sequencing. Multiple sequence alignment using BioEdit shows nucleotide sequences of TaqMan assay-amplified WC1 genes from cDNA relative to the reference gene sequence found in Genbank (see Table 1 for accession numbers). [file Image_2.TIF]

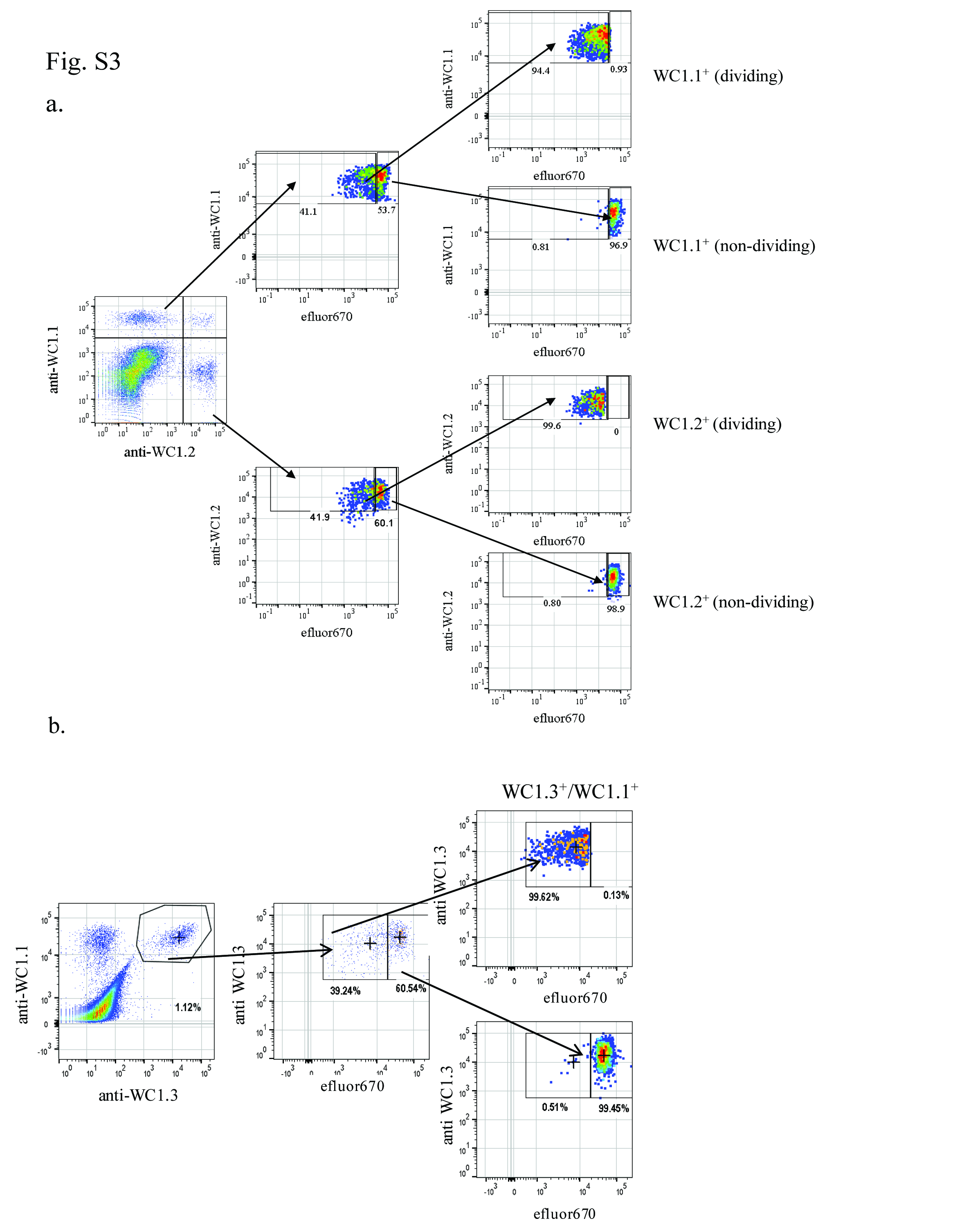

Supplement: Figure S3 — Sorting strategy to obtain WC1+ γδ T cell subpopulations for single cell cloning. (A) Single-positive WC1.1+ or WC1.2+ and (B) double positive WC1.1+/WC1.3+ γδ T cells were flow cytometrically analyzed and gates applied. The three gated cell populations were then evaluated for their level of cell division dye and the efluor-670low cells (indicative of multiple cell divisions) were collected as shown. This is representative of multiple flow cytometric sorts. [file Image_3.tiff]

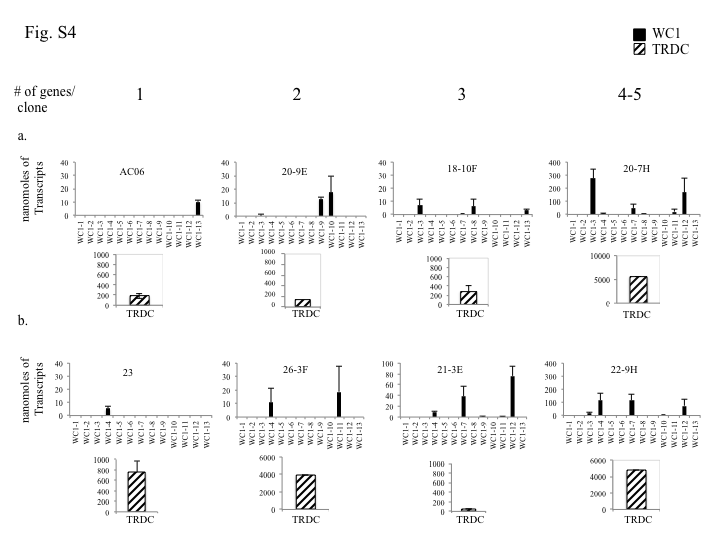

Supplement: Figure S4 — Representative clones with variable numbers of WC1 gene transcripts. Examples (from the 78 total clones) that had transcripts for one to five WC1 gene transcripts. If the mean was less than 2 and SE was at below zero, the gene was not included in the tally of transcripts in Figures 5 and 6 or Table 3. (A) WC1.1 cohort of γδ T cell clones from monoclonal antibodies (mAb) BAG25A+/CACTB32A− sorted cells expanded using expansion strategy 3 (Leptospira and IL-2) or (B) WC1.2 cohort of γδ T cell clones from mAb BAG25A−/CACTB32A+ sorted cells expanded with IL-2 with or without IL-15 and IL-18 supplementation. Moles of transcripts for each clone (mean ± SE) for WC1 and TRDC (hatched bars) are shown. [file Image_4.tiff]
